# Using CellML with OpenCMISS to Simulate Multi-Scale Physiology

**DOI:** 10.3389/fbioe.2014.00079

**Published:** 2015-01-05

**Authors:** David P. Nickerson, David Ladd, Jagir R. Hussan, Soroush Safaei, Vinod Suresh, Peter J. Hunter, Christopher P. Bradley

**Affiliations:** ^1^Auckland Bioengineering Institute, University of Auckland, Auckland, New Zealand; ^2^Department of Engineering Science, University of Auckland, Auckland, New Zealand

**Keywords:** CellML, OpenCMISS, physiome project, virtual physiological human, multi-scale physiological model

## Abstract

OpenCMISS is an open-source modeling environment aimed, in particular, at the solution of bioengineering problems. OpenCMISS consists of two main parts: a computational library (OpenCMISS-Iron) and a field manipulation and visualization library (OpenCMISS-Zinc). OpenCMISS is designed for the solution of coupled multi-scale, multi-physics problems in a general-purpose parallel environment. CellML is an XML format designed to encode biophysically based systems of ordinary differential equations and both linear and non-linear algebraic equations. A primary design goal of CellML is to allow mathematical models to be encoded in a modular and reusable format to aid reproducibility and interoperability of modeling studies. In OpenCMISS, we make use of CellML models to enable users to configure various aspects of their multi-scale physiological models. This avoids the need for users to be familiar with the OpenCMISS internal code in order to perform customized computational experiments. Examples of this are: cellular electrophysiology models embedded in tissue electrical propagation models; material constitutive relationships for mechanical growth and deformation simulations; time-varying boundary conditions for various problem domains; and fluid constitutive relationships and lumped-parameter models. In this paper, we provide implementation details describing how CellML models are integrated into multi-scale physiological models in OpenCMISS. The external interface OpenCMISS presents to users is also described, including specific examples exemplifying the extensibility and usability these tools provide the physiological modeling and simulation community. We conclude with some thoughts on future extension of OpenCMISS to make use of other community developed information standards, such as FieldML, SED-ML, and BioSignalML. Plans for the integration of accelerator code (graphical processing unit and field programmable gate array) generated from CellML models is also discussed.

## 1 Introduction

OpenCMISS (Bradley et al., 2011)^1^ is a general modeling environment that is particularly suited to biomedical engineering problems. It consists of two main parts: OpenCMISS-Zinc – a graphical and field manipulation library; and OpenCMISS-Iron – a parallel computational library for solving partial differential and other equations using a variety of numerical methods. It is a complete re-engineering of the CMISS (Continuum Mechanics, Image analysis, Signal processing, and System identification)^2^ computational code that has been developed and used for over 30 years.

The redevelopment of CMISS into OpenCMISS was driven by the desire to have an open-source project, to exploit modern parallel architectures, and to achieve a number of design goals unable to be met by the existing CMISS code-base. The first goal was that OpenCMISS would be a library rather than an application as CMISS was. This was to allow for OpenCMISS to be wrapped in an appropriate custom interface for clinical, educational, or commercial applications. The second goal was that the code should be as general as possible. Code or data structures that have been designed with too many assumptions may inhibit future applicability or when coupling problems.

The third goal was that OpenCMISS would be an inherently parallel code. Increasingly, complex or coupled models often require a parallel solution in order to decrease runtimes to acceptable levels. As computation codes often have lifetimes that are an order of magnitude greater than a particular parallel architecture OpenCMISS aims for a general heterogeneous parallel environment based on *n* × *p*(*n*) × *e*(*p*) computational units, where *n* is the number of distributed computational nodes, *p*(*n*) is the number of processing systems on the *n*^th^ computational node, and *e*(*p*) is the number of processing elements for the *p*^th^ processing system. Such a general parallel environment allows for multi-core or SMP systems, cluster systems, multi-core clusters and multi-core clusters with Intel Phi co-processors, graphical processing units (GPUs), field programmable gate array (FPGAs), or other hardware accelerators. OpenCMISS uses the MPI standard for distributed parallelism. There are currently research projects investigating the use of GPUs (using CUDA, OpenCL, and OpenAcc) and FPGAs for acceleration and the use of OpenMP for shared memory parallelism.

**Figure 1:**
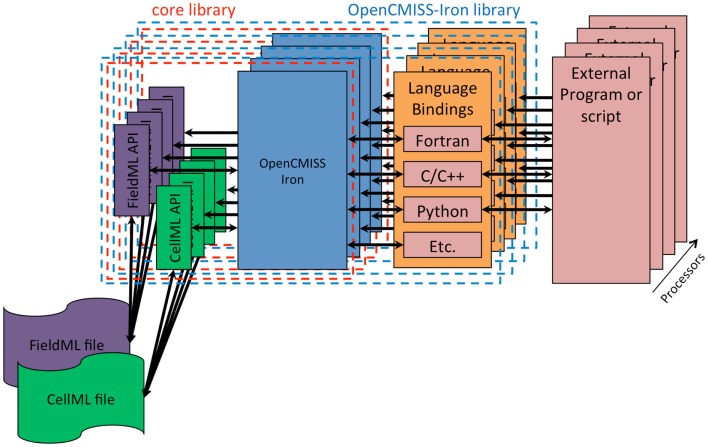
**OpenCMISS architecture diagram**. The external program or script is known as an *OpenCMISS application*, and makes use of the public OpenCMISS application program interface (API) via the most appropriate language bindings provided by the OpenCMISS library. The API itself then makes use of the internal core library to provide the required functionality. The core library makes use of the community provided CellML and FieldML library implementations to access data encoded in these standards.

The fourth design goal was that OpenCMISS should be used, understood, and developed by novices and experts alike. Modern scientific teams are often multidisciplinary in nature and thus team members can have very different backgrounds. The final design goal was that OpenCMISS should incorporate the Physiome Project (Hunter, 2004) markup languages FieldML (Britten et al., 2013) and CellML. The OpenCMISS architecture developed to achieve these design goals is shown graphically in Figure 1.

Here, we focus on the use of CellML to provide general purpose “plug and play” of mathematical models and model configuration in OpenCMISS applications. CellML (Cuellar et al., 2003)^3^ is an XML format for encoding mathematical models in a modular and reusable manner (Nickerson and Buist, 2008; Cooling et al., 2010). See Section 2 below for a general introduction to the mathematical framework provided by CellML. Also, in this Research Topic, see Hucka et al. (submitted) for an introduction to CellML and other related standards projects and Garny and Hunter (submitted) for one of the main integrated CellML software tools. OpenCMISS makes use of the CellML application program interface (API) (Miller et al., 2010)^4^ to interact with CellML models, and OpenCMISS-Iron defines a higher level CellML interface, which is then mapped to Fortran routines for use internally to the core library^5^.

## 2 Methods

The central data object in OpenCMISS is the field and models in OpenCMISS are defined using a collection of fields. The development of FieldML is closely aligned with this data model (Britten et al., 2013). The integration of CellML into OpenCMISS models and simulations is achieved using these fields. Therefore, we first introduce the key concepts underlying the field-based data model and then describe the integration of CellML with OpenCMISS models and simulations.

CellML is used in OpenCMISS applications for many different purposes. Following the above design goals for OpenCMISS, the actual implementation and usage of CellML is much more general than the previous implementation in CMISS (Nickerson et al., 2006). All applications using CellML in OpenCMISS follow a similar pattern. This can be seen in the examples described in Section 3 and the associated internet resources. The common application pattern is summarized here and described in more detail in the following sections.

1. Create a CellML Environment to manage a collection of models and their use.
2. Import CellML models into the CellML Environment.
3. Flag specific variables from each model as being relevant to the OpenCMISS model.
4. Map the flagged variables to fields.
5. Map variables from the CellML models to degrees-of-freedom (DOFs) in the OpenCMISS model.
6. Create OpenCMISS fields for the CellML variables that vary spatially.
7. Use the CellML environment in setting up the OpenCMISS model.
8. Define any solvers required in the evaluation of the CellML model(s).
9. Link the equations in the CellML model to the solvers.

### 2.1 OpenCMISS fields

Fields are the central mechanism in OpenCMISS for describing the physical problem and for storing any information required for this description. The comprehensive use of fields is a central concept of FieldML (Christie et al., 2009; Britten et al., 2013). OpenCMISS fields are hierarchical in nature. An OpenCMISS field contains a number of field “variables” and each field variable contains a number of field variable components. A field variable is thus equivalent to standard mathematical scalar, vector, or tensor fields.

Mathematically, a field is defined over a domain. In OpenCMISS, the conceptual domain for a field is the entire computational “mesh” (which could be a set of elements for some methods e.g., FEM, or a set of points for other methods e.g., meshless methods). However, in order to allow for distributed problems, the mesh is decomposed into a number of computational domains, which are each assigned to one computational node. Each computational node only allocates and stores information for its domain and any fields defined over that domain.

OpenCMISS allows for each field variable component to have a different structure for its DOFs. Structures that are currently supported are: constant structure (one DOF for the entire component); element structure (one or more DOFs for each element); node structure (one or more DOFs for each node); Gauss point structure (one or more DOFs for each Gauss or integration point); and data point structure (one or more DOFs for each data point). In addition, for node structures, which are used for standard finite element type interpolation, OpenCMISS allows for each element to have a different basis function.

OpenCMISS collects all DOFs from all the components in a field variable and stores them as a distributed vector. The DOFs stored in the distributed vector include those from the computational domain and a layer of “ghosted” DOFs (local copies of the value of DOFs in a neighboring domain). To ensure consistency of data OpenCMISS handles the updates between computational nodes if a node changes the value of a DOF, which is ghosted on a neighboring computational node.

### 2.2 Mathematical framework

In general, CellML models describe a vector system, **F**, of differential-algebraic equations (DAEs) of the form:

(1) $$F\left(t,x,\mathrm{x\prime},a,b\right)\;=\;0,$$

where *t* is the independent variable, x is a vector of state variables, x′ is a vector of the derivatives of state variables with respect to the independent variable, a is a vector of independent parameters, and b is an optional vector of intermediate “output” variables from the model (i.e., derived from the other variables but does not affect the system of equations).

CellML models are typically used for processes that occur at an abstract point in space, i.e., for a particular spatial scale of interest the processes can be considered to occur in a region of space small enough to be considered a point and are thus known as zero-dimensional (0D) models. Whilst 0D models are useful, there are numerous applications of interest that occur in higher dimensions. In order to use CellML models in multi-scale, multi-dimensional models we require a method which can: (a) locate a CellML model at a particular spatial location; (b) allow the 0D CellML model variables to affect the spatial fields of variables of the higher dimensional models; and (c) allow the values of the higher spatial dimensional field variables at the location of the 0D CellML model to affect the CellML model variables.

In numerical methods, the higher dimensional fields are often interpolated in some manner. Interpolation can be thought of as calculating the value of a field at some location in its domain by using some mathematical functions (interpolation or basis functions) operating on a set of numerical values (the DOFs). The interpolation functions are chosen based on the numerical method being used and modeling decisions of the modeler. Once the interpolation functions have been fixed then the value of a field is determined by its DOFs. Control of the DOF values is thus a good candidate to allow 0D models to affect the values of the spatial fields.

**Figure 2:**
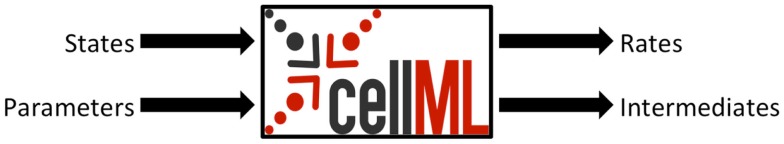
**The CellML black box model showing state and parameter variables as inputs and rate and intermediate variables as outputs**. Such a black box is designed as a general model evaluation object, which can plug into a variety of numerical methods and workflows. An ODE-type model might, for example, plug into an integration solver to simulate the evolution of the model over time. Whereas, a pure algebraic model would not have any state variables and pure evaluation solver is able to directly compute the intermediate variables from a given set of input parameters.

In OpenCMISS, a CellML model is considered to be a black box model for the value of a DOF. As shown in Figure 2, the black box model has two inputs and two outputs. The inputs are the state variables, x, and the parameter variables, a, and the outputs are the rate variables, x′, and the intermediate variables, b.

### 2.3 CellML environment

The main object within OpenCMISS for managing CellML models is the “CellML environment” container object. Once an environment object has been started the next step is to import required CellML models into the environment from specified XML files. Multiple CellML models can be imported into one CellML environment and multiple CellML environments can be used in a given OpenCMISS application. To distinguish between the models within an environment an integer model index is returned from each import and this index can subsequently be used to reference the CellML model in OpenCMISS.

The CellML environment is distributed over all computational domains in the OpenCMISS application. CellML models imported into the environment are therefore available on all computational nodes (independently).

### 2.4 Flagging CellML model variables

As described above, it is important in multi-scale models that CellML variables can influence the higher dimensional field variables and vice-versa. It should be noted, however, that for some models, not all the CellML variables interact with the field variables. For example, it may be the case that a certain parameter to the CellML model does not vary spatially. The user is able to flag each CellML variable as either “known” and/or “wanted.” If a variable is known then its numeric value will be controlled by a field in OpenCMISS – i.e., the variable is passed into the CellML black box model (states or parameters in Figure 2). If a variable is wanted then its numeric value computed by an evaluation of the CellML model will be used outside of the CellML model – i.e., the variable will be passed out of the CellML black box model (rates or intermediates in Figure 2). When importing a model, the default behavior in OpenCMISS is that all state variables and the independent variable are flagged as known and wanted; no other variables have any flags set.

Once the desired CellML variables have been flagged the construction of the CellML environment can be finished – OpenCMISS now has enough information from the application to determine which variables in the CellML models require exposure to the fields. Finishing the CellML environment means that each CellML model can be instantiated into a computable black box. When a model is instantiated, the CellML API (Miller et al., 2010) is used to generate a procedural representation of the model to determine which CellML variables are free (they are either known or wanted) and which variables are fixed (no flags set). The code generation service of the CellML API then generates a computer code function for use in evaluating the model. The function has a standard interface, e.g., for C code:

void CellML_routine(double VOI, double* STATE, double* RATE, double* KNOWN, double* WANTED);

which is of the form of Equation 1. Here, VOI is the independent variable, *t*; STATE is the vector of state variables, x; RATES is the vector of derivatives, x′; KNOWN is the vector of parameter variables, a; and WANTED is the vector of intermediate variables, b. Variables in the CellML model that are fixed are not passed as parameters to the generated CellML routine. Instead, they are set as constants in the generated computer code with their value given by the CellML model.

### 2.5 Field maps

**Figure 3:**
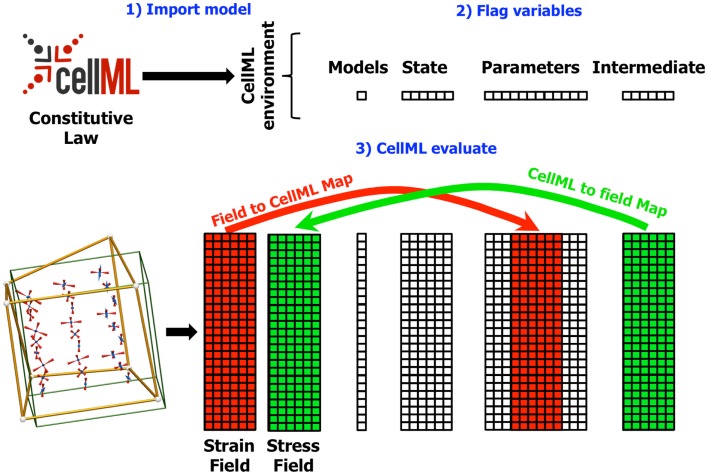
**Diagram showing the field maps for a finite elasticity example similar to that described in Section 4**. In this example, the components of the strain field in the OpenCMISS model are mapped to CellML variables in the CellML parameters field. Similarly, the CellML variables in the intermediate field representing the components of the stress tensor are mapped back to the stress field in the OpenCMISS model. The mapping is defined once the CellML model has been imported (1) and the required variables have been flagged (2). Whenever the CellML model is evaluated (3), the values from the strain field are first transferred to the CellML parameters field, the model is evaluated, and the values from the CellML intermediate fields are transferred back to the stress field.

The next step in using a CellML model in OpenCMISS is to define the field maps. These maps link CellML variables with OpenCMISS field variable components. There are two types of maps depending on the direction of data flow, as shown in Figure 3. A field to CellML map links the component of an OpenCMISS field with a known CellML variable. A CellML to field map links a wanted CellML variable with a component of an OpenCMISS field variable. The field maps are specified by identifying a particular component of an OpenCMISS field variable and the name of a CellML variable of a CellML model that has been loaded into the CellML environment. In addition to linking variables the field maps also determine the DOF “pattern” or image of the CellML models. OpenCMISS looks at each DOF in each component of an OpenCMISS field that has been mapped and determines the DOF location (e.g., the position of the node, Gauss point, data point, etc. corresponding to the DOF). These locations then serve as the geometric positions of the CellML models – i.e., conceptually there is an instance of a CellML model located at each DOF location. The field maps are checked to ensure that the DOF locations for OpenCMISS field variable components that are mapped to each CellML variable for a particular CellML model are compatible. To be compatible all CellML variables in a model must be mapped to OpenCMISS field variables that have their DOFs at the same locations. Note that this does not mean they must be mapped to the same DOFs just that the DOFs must be located at the same point in space. For example, in a standard finite element type field where the DOFs are located at node points, different components of an OpenCMISS field variable could be mapped to CellML variables or different components from different field variables could be mapped provided the different field variables had the same interpolation (basis).

### 2.6 CellML fields

Once the field maps have been defined and the DOF pattern of CellML models determined the next step is to define CellML fields. The CellML fields are standard OpenCMISS fields, which are used to store values of the spatially varying CellML variables. There are four different types of CellML fields (shown in Figure 3) – a models field (see below), a state field, which stores the CellML model state variables, a parameters field, which stores the CellML parameters variables, and an intermediates field, which stores the CellML intermediates variables. The CellML field DOF values can be conceptually thought of as an array with the number of rows equal to the number of CellML models (one at each DOF in the pattern) and the number of columns equal to the number of CellML variables in each model, i.e., each row of the array corresponds to the values of the CellML variables for one particular CellML model.

The CellML fields allow for a spatial variation in the value of any CellML variable. As the CellML fields are standard OpenCMISS fields, the exact form of the spatial variation is determined by the choice of interpolation and the values of the CellML field DOFs. The default value and variation of each CellML state, parameter and intermediate variable is given in the CellML XML file and is constant across the domain. OpenCMISS also allows for a spatial variation of the actual CellML model. The CellML models field is an integer-valued field, which can be used to specify which CellML model in the CellML environment is used at each DOF in the pattern. The default choice is the first model loaded into the environment but other models can be selected by setting the value of the models field DOF to the value of the model index returned when importing the model. Setting the models field to zero at a particular DOF in the pattern will result in no CellML model at that particular DOF.

When setting up their models and simulations, OpenCMISS users are able to take advantage of some internal OpenCMISS memory optimizations. If a user chooses matching DOF patterns in different parts of their model definition, they are able to simply use existing fields in place of the CellML fields. In this case, rather than duplicating internal storage for the fields and copying values between the fields, the data arrays are able to be used directly.

### 2.7 Solvers

**Figure 4:**
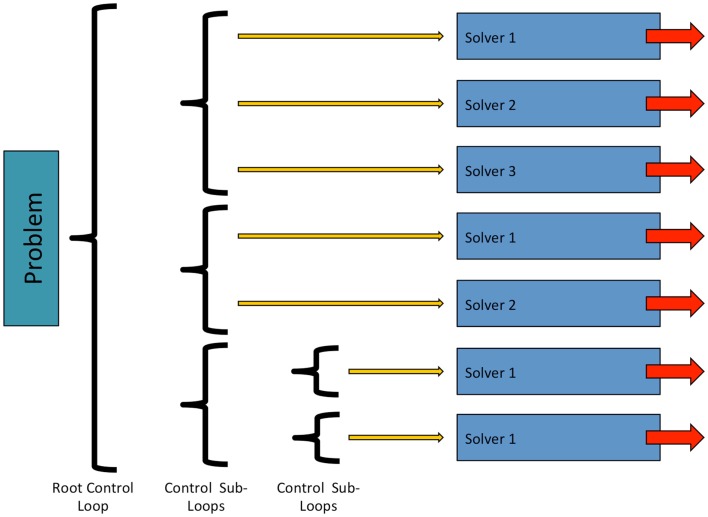
**Structure and relationship between OpenCMISS control loops and solvers**. See Figure 6 for an example showing how an OpenCMISS application will use these workflow capabilities.

In OpenCMISS, solvers are objects, which perform numerical “work” as part of some problems workflow. This numerical work is not restricted to that of traditional solvers such as linear or non-linear solvers, and can take other forms, e.g., translation and rotation of a mesh. As shown in Figure 4, solvers are contained within a control loop that has no sub-loops. Each control loop can contain an arbitrary number of solvers. When a control loop is executed each solver is executed in turn. The ability to nest control loops provides a mechanism to have different time scales for different models. For example, in Section 3 we present simulations including a cardiac electrophysiology example. For this model, we can “solve” the CellML model of a cardiac cell at a much finer time scale than the “solve” of the reaction diffusion model.

OpenCMISS has two CellML specific solvers implemented. The first solver is a CellML evaluation solver. When this solver is executed each CellML model at each DOF is executed. The second solver is a CellML integration solver. When this solver is executed it integrates the equations in a CellML model from a specified start time to a specified stop time.

In addition to constructing a workflow using solvers within control loops, a workflow may be created by linking a solver to another solver. An example of solver linking occurs when a Newton type non-linear solver links to a linear solver. The linear solver is then used to compute the search direction as part of a major Newton step of the non-linear solvers iterations. CellML solvers may also be linked to other solvers. An example of when this is used is when CellML evaluation solvers for computing the stress state using a constitutive law in large deformation mechanics. These mechanics simulations are non-linear and are typically solved in a manner in which residual equations are repeated evaluated by a non-linear solver. By linking a CellML evaluation solver to the non-linear solver the constitutive law can be evaluated for the state of deformation given by the solver as part of the residual evaluation.

In summary, the simulation process for using CellML with OpenCMISS is as follows: OpenCMISS starts the execution of a problem by looping through the top level control loop. Sub-control loops are looped through in turn until a loop with solvers is encountered. The solvers are then executed in turn. If the solver is a CellML solver then the solve starts by transferring the current value of mapped OpenCMISS fields to the corresponding CellML fields. The CellML solver is then executed. After the CellML solver has finished the value of the CellML fields that are mapped are transferred to OpenCMISS fields.

## 3 Results

We have provided some documented examples of OpenCMISS applications, which demonstrate the capabilities of using CellML models with OpenCMISS-Iron. These are available at: http://opencmiss-cellml-examples.readthedocs.org/. The documentation provides links back to the free and open-source driving these applications on GitHub^6^. In the following sections, we provide brief introductions to the example applications available and highlight some of the multi-scale and multi-physics abilities of OpenCMISS and CellML. More complete details are available at the above internet location.

### 3.1 Basic usage

The “OpenCMISS-Iron CellML Examples” provide an introduction to the common usage of the OpenCMISS-Iron API relating to the use of CellML models as described in Section 2. The examples here do not define complete models or numerical simulations, but rather demonstrate the basic initialization steps required regardless of the actual application being developed. In this section of the online supplement, we provide the same example application using the two most common language bindings for OpenCMISS-Iron, namely Fortran and Python.

### 3.2 Cardiac electrophysiology

To illustrate the use of CellML in a more physiologically applicable example, the monodomain equation (Keener and Sneyd, 1998) is solved in a square 2D domain using a CellML electrophysiology model obtained from the CellML model repository. When modeling electrophysiology, two of the most common modeling variations are the choice of the particular cell model and a spatial variation of material and cellular parameters (e.g., when, say, modeling an infarct). The use of CellML allows a modeler to use any electrophysiology cell models that can be represented in CellML without having to change the numerical simulation code. The linking of CellML variables to OpenCMISS fields allows cellular and material parameters, alike, to be easily varied in complex ways.

**Figure 5:**
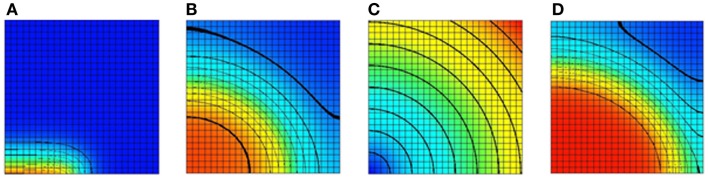
**Results of a 2D monodomain solution with a Noble 98 ventricular cell model**. **(A)** A plot of the transmembrane voltage immediately after a stimulus along half of the bottom edge. **(B)** A plot of the transmembrane voltage after a fixed time with an isotropic value of the sodium channel conductance gNa. **(C)** A spatial variation of gNa. The sodium channel conductance is varied from its normal value at the bottom left node to 300% of its normal value at the top right node. **(D)** A plot of the transmembrane voltage with a varying sodium channel conductance after a fixed period. Comparing with **(B)** it can be seen that increasing the sodium channel conductance increases the conduction velocity. For **(A,B,D)** the value of the transmembrane voltage varies from −95 mv (blue) to +50 mV (red).

The monodomain equation is often solved using an operator splitting approach (Qu and Garfinkel, 1999; Sundnes et al., 2005). In this example, a Gudunov split is used to break the monodomain equation into an ordinary differential equation (ODE) and a parabolic equation. The model domain in this example consists of a square domain divided into 25 elements in each direction. The tissue conductivity is isotropic. Bilinear Lagrange finite elements are used in the solution of the parabolic problem. For the ODE problem, a Noble 98 guinea-pig ventricular model (Noble et al., 1998) is attached to each node in the domain. A stimulus current was applied to the leftmost half of bottom row of nodes. A plot of the transmembrane voltage immediately after the stimulus current was turned off is given in Figure 5A.

To illustrate the ability to spatially vary CellML parameters two simulations were performed. In the first simulation, the sodium channel conductance, gNa, was isotropic and left at its normal value. A plot of the transmembrane voltage after a fixed time is shown in Figure 5B. In the second simulation, the sodium channel conductance was varied in a radial pattern as determined by the distance from the bottom left node. The channel conductance was varied from 100% of its normal value at the bottom left node to 300% of its normal value at the top right node. A plot of the spatial distribution of sodium channel conductance is shown in Figure 5C. A plot of the transmembrane voltage in the second simulation after the same fixed time period is shown in Figure 5D. Comparing Figure 5B with Figure 5D it can be seen that in second simulation the activation wave front has advanced further into the domain for the same fixed time period. This shows that increasing the sodium channel conductance increases the activation wave front conduction velocity.

### 3.3 Fluid dynamics boundary conditions

Constructing full subject-specific computational fluid dynamics (CFD) models of the entire arterial and/or venous vasculature is currently considered impractical, owing to: (1) the time and resources required to identify, segment, and constrain a model of the billions of vessels in a human body; and (2) the computational cost such a model would incur.

However, as blood flow is primarily driven by the pressure gradients between the heart and downstream vascular beds, a modeled vessel must still be considered within its systemic context to be physiologically relevant. This can be accomplished by coupling simpler, lumped-parameter/0D models to the more computationally expensive (3D/1D) CFD models at domain boundaries. This involves coupling together dependent fields (i.e., pressure and velocity/flow), material fields (e.g., fluid viscosity and wall compliance), and geometric fields (e.g., vessel diameter) at the interfaces between 3D, 1D, and/or 0D model domains.

In the “Fluid Mechanics: Navier–Stokes: 1D-0D Visible Human Example,” a 1D network of 24 major arteries is constructed from the male Visible Human dataset (reproduced in Figure 7). Over this domain, the 1D formulation of the Navier–Stokes equations and its Riemann invariants are solved for flow rate and pressure. Flow rate from a published dataset is applied at the aortic root to provide inlet boundary conditions. At each of the terminal (outlet) boundaries of the 1D domain, a 0D RCR Windkessel model is applied to approximate downstream vascular impedance.

**Figure 6:**
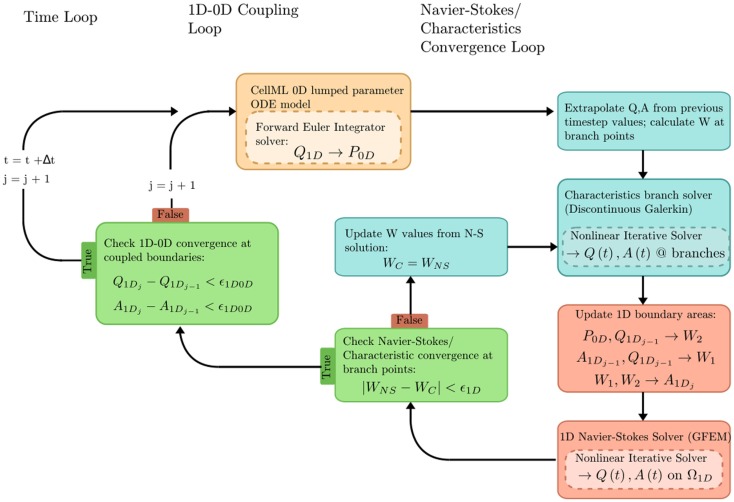
**Overview of the coupled 1D–0D solution process, which is defined in our fluid dynamics boundary conditions example**.

The problem solution workflow for this example is depicted in Figure 6. Flow rate (Q) from the 1D OpenCMISS solver provides the forcing term for the CellML ODE solver. Pressure (P) is returned from CellML to provide constraints on the Riemann invariants of the 1D system, which translate to area boundary conditions for the 1D solver. At each timestep, the 1D and 0D systems are iteratively coupled until the boundary values converge within a user-specified tolerance at the 1D–0D interfaces.

**Figure 7:**
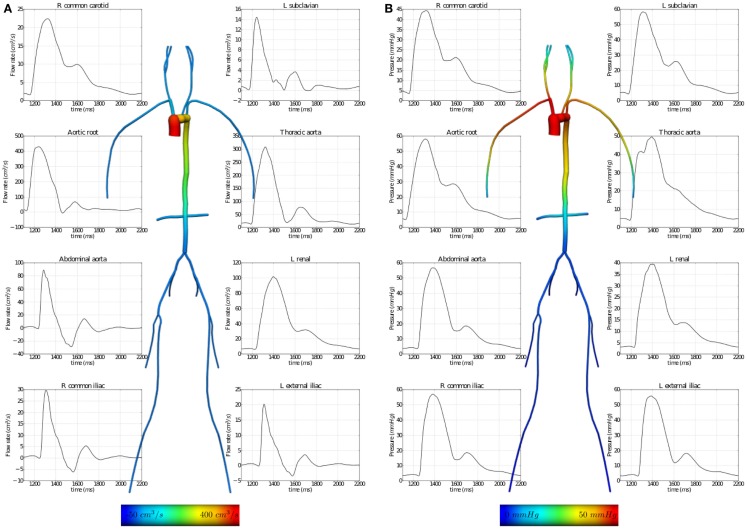
**Results from executing the fluid dynamics boundary conditions example OpenCMISS application**. **(A)** The distribution of flow rates and **(B)** that of fluid pressures within the vessel segments. The graphs illustrate the temporal variation during one cardiac cycle and the vessel images are a temporal snapshot at peak systole.

The results of executing this example OpenCMISS application are shown in Figure 7.

Other applications of OpenCMISS and CellML coupling for fluids include coupling of 3D and 0D models and hemorheological constitutive laws that approximate the shear-thinning behavior of blood.

### 3.4 Mechanical constitutive laws

As described previously, a common case for the usage of CellML models in OpenCMISS applications is to specify mechanical constitutive laws (the relationship between strain and stress) in finite elasticity applications. By using CellML models to describe the constitutive laws required for a given finite elasticity model, the implementation of the equations governing finite elasticity are able to be generic without needing specific relationships to be “hard-coded” in the core OpenCMISS library. This clearly aligns with the design goals for OpenCMISS.

The “Axial extension in a homogeneous pipe” example demonstrates how a CellML model can be used to define the mechanical constitutive law for a finite elasticity OpenCMISS application. In this example, a homogeneous cylinder (a blood vessel, for example) is stretched along its longitudinal axis. The Mooney–Rivlin constitutive law (Rivlin and Saunders, 1951) is used in this example, but by importing a different CellML model the user would be able to change the behavior of this application.

**Figure 8:**
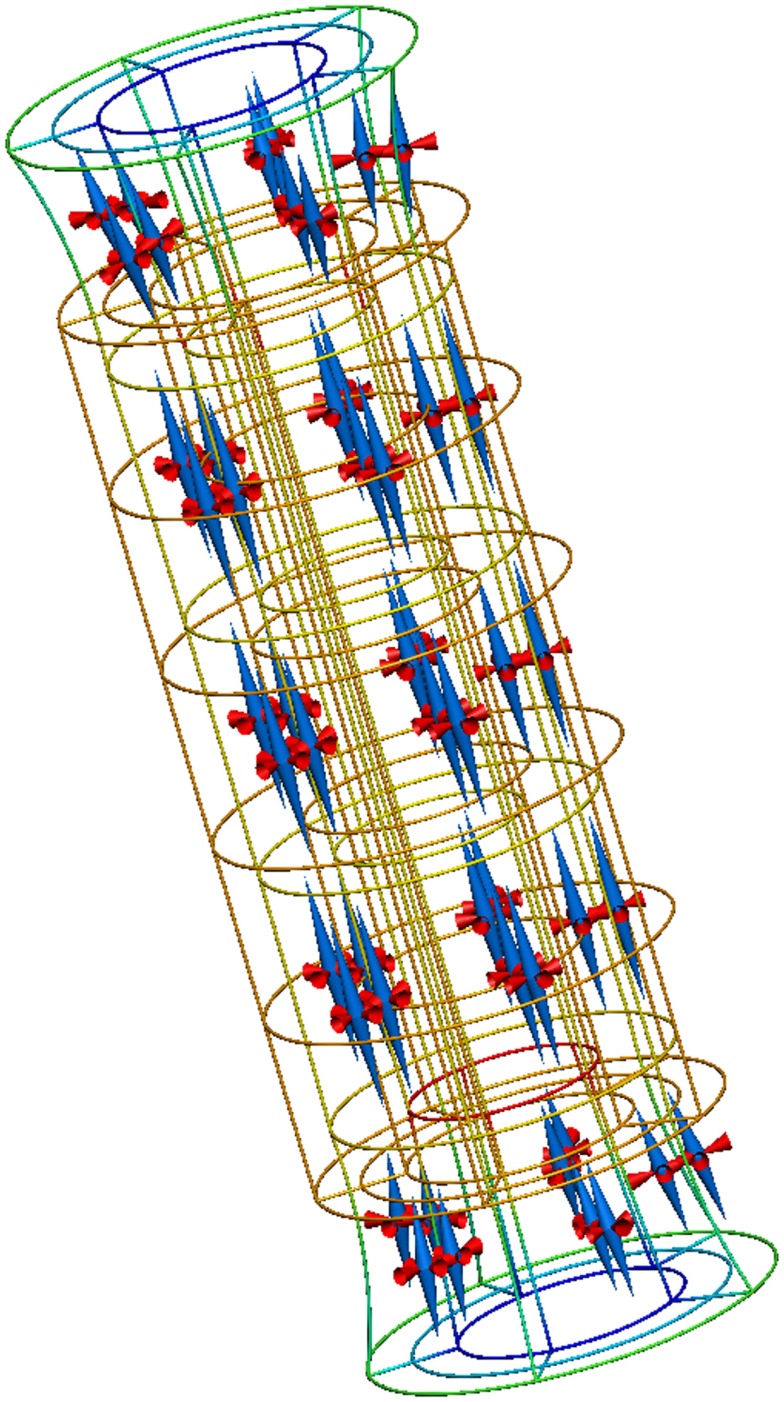
**Results from running this cylinder extension application**. The gold lines show the original, undeformed, cylinder geometry. The colored lines show the deformed geometry, with the color varying to show the difference in strain through the wall of the cylinder. The cones represent the three normalized principal strains at material points throughout the tissue volume (red for compression and blue for extension).

A complete description of this example is available from the internet location referenced above and Figure 8 reproduces the results from executing this example application.

## 4 Discussion and Conclusion

We have presented the methods by which CellML is used in OpenCMISS to provide a very flexible “plug and play” system for users to leverage when creating OpenCMISS applications. The examples presented in Section 3 are not meant to present novel findings, but rather demonstrate the implementation of OpenCMISS applications, which take advantage of this system to exemplify the underlying design goals for OpenCMISS.

Encapsulating aspects of the OpenCMISS model and simulation in CellML models not only allows interoperability with other tools, which support CellML (Garny et al., 2008; Beard et al., 2009), but also enables the exploration of various computational optimizations. Some of these optimizations are generic for any CellML model see for some discussion on potential optimizations (Garny et al., 2008) and others make use of the high-performance computing environments in which OpenCMISS is designed to be used.

For certain simulations in OpenCMISS, a CellML model can be evaluated a very large number of times resulting in a significant computational time. In order to reduce this time, we can take advantage of the fact that each instance of a CellML model at a particular DOF is completely independent from CellML models at every other DOF and evaluate the models in parallel. The framework for OpenCMISS and CellML presented in this paper involves a small number of CellML models each involving a short fixed portion of code executing a large number of times with different data and is ideally suited for hardware acceleration with GPUs and FPGAs. Work is currently underway on updating our framework so that instead of generating C code from the CellML model, GPU code (CUDA, OpenCL, or OpenAcc) is generated with the CellML model forming the computational “kernel.” Work on using CellML with FPGAs has also started with the aim of generating VHDL (VHSIC Hardware Description Language) code from the CellML model (Yu et al., 2013).

To further improve the interoperability of OpenCMISS with other software tools and user communities, we are actively pursuing a broader range of support for community standards. CellML itself is one of the core COMBINE standards (Hucka et al., submitted) and we are considering how to best use the other standardization efforts under the COMBINE consortium. The simulation experiment description markup-language SED-ML; (Waltemath et al., 2011) is an obvious candidate for use in OpenCMISS. As an initial step toward adopting SED-ML, the CellML solver configuration for a given simulation could be defined using SED-ML. Further work in contributing to the evolution of SED-ML to enable the encoding of complete OpenCMISS simulation descriptions in future versions of SED-ML is also being considered. This could potentially build on top of recent developments in the area of functional curation (Cooper et al., 2011).

As mentioned previously, the OpenCMISS field-centric data model is a major driving factor in the development of FieldML (Christie et al., 2009; Britten et al., 2013). In order to be able to fully and unambiguously describe the full range of fields available in OpenCMISS, further work is required to expand the capabilities of FieldML, both in terms of the standard itself and its supporting software library. Another proposed standard that is closely related to both CellML and FieldML is BioSignalML (Brooks et al., 2011). BioSignalML is a proposed standard for the description of temporal physiological signals and could be used in OpenCMISS to describe time-varying boundary conditions either directly applied to the OpenCMISS model or to the CellML models used in an OpenCMISS application.

OpenCMISS has been, and continues to be, developed as a high-performance computational platform aimed at large-scale physiological modeling. As such, OpenCMISS simulations are generally limited by the computational hardware available (memory, storage requirements) and the acceptable duration of a simulation, rather than any inherent limitation in the software code itself. In addition to the specialized hardware developments mentioned above, current work involves the building and execution of OpenCMISS simulations on some of the largest computers available in the world. While we expect the linkage between OpenCMISS and CellML models to work as described above, unexpected issues may arise during the porting of software to such large machines. Any such issues will need to be addressed on a case-by-case basis and require the expertise of various hardware and compiler specialists with whom we collaborate.

One limitation of our current approach is that it is sometimes necessary to copy the data for each CellML model either between fields or to temporary memory. This is to ensure a contiguous layout of a CellML models data for optimal evaluation. This is particularly so for simulations that use a number of different CellML models at different DOFs. It may be possible to avoid this situation if the multiple CellML models can be combined into one model with the switch on sub-models occurring inside the one CellML model, possibly based on a combination of OpenCMISS field values. This work-around would result in a higher total memory overhead but a reduction in computational cost.

The combination of OpenCMISS and CellML provides a powerful tool for users to customize a very general computational physiology software library to meet their specific application requirements. In this manner, we are close to achieving the stated goals driving the development of OpenCMISS. Through the use of CellML, and other standards in the future, OpenCMISS is able to be a general-purpose library, which can be wrapped in the appropriate custom interface for a wide range of applications. By abstracting the computational details in the OpenCMISS library and providing the ability to use CellML, users are able to make use of a range of tools to create, edit, and interact with their CellML models e.g., (Garny and Hunter, submitted), thus enabling novice users to relatively easily develop complex OpenCMISS applications.

## Author Contributions

David P. Nickerson, Christopher P. Bradley, and Peter J. Hunter conceived, designed, and implemented CellML support in OpenCMISS. All authors contributed to the demonstration examples and this manuscript. All authors contribute to the development of OpenCMISS.

## Conflict of Interest Statement

The authors declare that the research was conducted in the absence of any commercial or financial relationships that could be construed as a potential conflict of interest.

## Footnotes

^1^http://www.opencmiss.org/

^2^http://www.cmiss.org/

^3^http://cellml.org/

^4^http://cellml-api.sourceforge.net/

^5^http://github.com/OpenCMISS/cellml/

^6^http://github.com/
